# The survival of non-traumatic osteonecrosis of femoral head at ARCO II with ring-shaped sclerotic zone: a mid-term follow-up retrospective study

**DOI:** 10.1093/jhps/hnab013

**Published:** 2021-03-26

**Authors:** Zhong-Shu Wu, Guoju Hong, Peng Yang, Fan Yang, Zhen-Qiu Chen, Wei He, Qiu-Shi Wei

**Affiliations:** 1 First Clinical Medical College, Guangzhou University of Chinese Medicine, Guangzhou 510405, China; 2 Division of Orthopaedic Surgery, The University of Alberta, 116 Street, 85 Avenue, Edmonton, AB T6G 2R3, Canada; 3 No. 3 Orthopaedic Region, The First Affiliated Hospital of Guangzhou University of Chinese Medicine, Guangzhou 510405, China; 4 Department of Orthopaedics, The Third Affiliated Hospital of Guangzhou University of Chinese Medicine, No. 261 Longxi Road, Liwan District, Guangzhou 510378, China; 5 Traumatology and Orthopedics Institute of Guangzhou University of Chinese Medicine, No. 261 Longxi Road, Liwan District, Guangzhou 510378, China

## Abstract

The sclerotic zone in the osteonecrosis of femoral head (ONFH), containing condensed trabecular bone and abundant neovascularization, is the transition area between osteonecrosis and normal tissue. Due to the prominent feature in ONFH, the characteristics of the sclerotic zone might indicate the femoral head survival of the disease. Thirty ONFH patients (41 hips) with ring-shaped sclerotic zone at Association Research Circulation Osseous-II were recruited during 1996 to 2019, and the corresponding radiographic images in their follow-up are reviewed retrospectively. Two subtypes (type A and B) are defined to discriminate different locations of ring-shaped sclerotic zone in the femoral head (center or subchondral bone plate) in accordance with the radiographic images. The natural history of the enrolled subjects was followed up for average 9 years to record and compare their collapse incidences as well as the progress of hip symptoms. Chi-square test shows that the occurrence rates of symptomatic hip of type A are significantly lower than that of type B and differences between these two groups were significant (*P* < 0.05). Kaplan Meier survival curve analysis shows that the mean survival time of type A is 247.600 M (95% CI: 203.072 ∼ 292.128 M) and type B is 88.795 M (95% CI: 72.607 ∼ 104.984 M). The survival rate of femoral head of type A is significantly higher than that of type B (*P* < 0.005). This study demonstrates that type A shows a more satisfactory clinical outcomes and lower femoral head collapse rate in a mid-term follow-up.

## INTRODUCTION

Non-traumatic osteonecrosis of the femoral head (ONFH) commonly occurs in young and middle-aged adults and the prevalence has been reported to be increasing [1, 2]. Non-traumatic ONFH is a potentially devastating disease that often leads to collapse of the femoral head and arthritis of the hip [3–5]. One of the main factors affecting the prognosis of ONFH is femoral head collapsing. Current researches indicate that most of the cases collapse within 1–4 years after diagnosis [6–8]. Then arthritis follows, the joint pain and dysfunction seriously affects people’s daily life, eventually, joint replacement is unavoidable [9–12]. Moreover, since this disease is common in young people [13] and the joint prostheses are not durable [14, 15], the artificial joints replacement procedures subject to be revised repeatedly, causing great pain and increasing economic burden. Therefore, many studies focused on the methods to predict the collapse of ONFH. The past researches have showed the size and location of the lesion are the most important risk factors for symptom development. The necrotic type with small size and middle location has a better prognosis [16–18]. Other researches describe the case with recognizable necrotic foci within the femoral heads and a normal contour of the weight-bearing surface present no symptoms and normal hip function [19–21]. Furthermore, a research point that a type which necrotic foci present a round, cystic appearance circumscribed by radiodense lines on anteroposterior roentgenograms may need no treatment or less aggressive treatment than other types [22]. Currently, many studies have tried to predict the collapsing of ONFH, but most focus on the necrotic region [23, 24].

The sclerotic zone is a symbol of bone repair after ONFH, containing with condensed trabecular bone and abundant neovascularization, is the transition area between osteonecrosis and normal tissue [25–28]. On the radiographic films and computed tomography (CT) scans of patients with ONFH, there is an area surrounded by the rim of irregular mottling or a striped high-density shadow. Most researches [29–33] show that the formation of sclerotic zone can delay the collapse of ONFH. When ONFH occurs, the ability of osteoblasts to produce new bone and osteoclasts to remove dead bone is not balanced [34, 35]. At the same time, bone trabecular fracture leads to the decline of bone mechanics during bone repair [36] and even the collapse of the femoral head. The condensed trabecular bone in the sclerotic zone protect necrotic tissue [29], maintain the mechanical strength during the repair process [30, 31] and provide the structural integrity of the femoral head [28, 30], and thus played a role in preventing collapse. Under certain conditions, the closed sclerosis rim delaying collapse, by reducing the original high stress in the subchondral bone, without it the collapse rate is as high as 76% [25, 32, 33]. One study shows when the sclerotic rim proportion is >30%, the risk of collapse is low, and the prognosis of ONFH is satisfactory [37]. However, a few researchers debate that the sclerotic rim may hinder the growth of neovessels and newly generated tissue toward the necrotic tissues and is not conducive to bone repair [38–41].

In the study of natural history of ONFH, sclerotic zone’s role has not been paid enough attention, and the specific mechanism is not clear. Hence, the goal of this retrospective study is to report the prognosis of the Association Research Circulation Osseous (ARCO) stage-II ONFH with ring-shaped sclerotic zone.

## MATERIALS AND METHODS

### Patients

ARCO stages [42] are used for classification of osteonecrosis (Tables I and II). Our study is approved by ethical board of the First Affiliated Hospital of Guangzhou University of Chinese Medicine. From 1996 to 2019, we regularly requested each patient for magnetic resonance imaging (MRI), anteroposterior view and frog-leg lateral view radiographs to assess the progress of the ONFH. We have retrospectively reviewed 30 ARCO stage II patients (41 hips) with ring-shaped sclerotic zone, and in whom follow-up was possible for at least 64 months. There are 22 male and 8 female patients, the mean age is 47 years (range, 27–71 years), and the mean follow-up is 104 months (range, 64–273 months). The osteonecrosis is associated with steroid therapy, alcohol abuse or both, in 21 patients (70%) and is idiopathic in 9 (30%). In the 41 hips, there are 30 cases of type A and 11 cases of type B.

**TABLE I.** Data on the 32 hips who had not collapsed at the most recent follow-up

Hip number	Patient number	Gender, Age (years)	Associated condition	ARCO		Symptom at onset	Follow-up time (Months)	Ficat stage at follow-up	Comments
				Stage at onset	Type				
1	1	F, 41	Steroid use	L: II	A	L: asymptomatic	111	L: II	L: asymptomatic
2	2	M, 48	Steroid use, alcohol abuse	L: II	A	L: asymptomatic	69	L: II	L: asymptomatic
3	3	M, 46	Steroid use	R: II	A	R: asymptomatic	165	R: II	R: asymptomatic
4	5	M, 51	Steroid use, alcohol abuse	R: II	A	R: asymptomatic	117	R: II	R: asymptomatic
5	6	M, 50	Idiopathic	L: II	A	L: asymptomatic	127	L: II	L: asymptomatic
6	6	M, 50	Idiopathic	R: II	A	R: pain	127	R: II	R: asymptomatic
7	7	M, 33	Idiopathic	L: II	A	L: asymptomatic	75	L: II	L: asymptomatic
8	7	M, 33	Idiopathic	R: II	A	R: pain	75	R: II	R: asymptomatic
9	8	F , 57	Steroid use	L: II	A	L: asymptomatic	116	L: II	L: asymptomatic
10	8	F, 57	Steroid use	R: II	A	R: asymptomatic	116	R: II	R: asymptomatic
11	9	F, 48	Idiopathic	L: II	A	L: asymptomatic	150	L: II	L: asymptomatic
12	10	M, 62	Steroid use	R: II	A	R: asymptomatic	88	R: II	R: asymptomatic
13	13	M, 48	Alcohol abuse	R: II	A	R: asymptomatic	76	R: II	R: asymptomatic
14	14	M, 28	Idiopathic	L: II	A	L: asymptomatic	113	L: II	L: asymptomatic
15	15	M, 27	Steroid use	R: II	A	R: asymptomatic	80	R: II	R: asymptomatic
16	16	M, 35	Idiopathic	L: II	A	L: asymptomatic	85	L: II	L: asymptomatic
17	17	M, 46	Steroid use	R: II	A	R: asymptomatic	130	R: II	R: asymptomatic
18	19	F, 32	Steroid use	L: II	A	L: pain	92	L: II	L: asymptomatic
19	20	M, 47	Idiopathic	L: II	A	L: pain	113	L: II	L: asymptomatic
20	22	M, 54	Idiopathic	R: II	A	R: discomfort	77	R: II	R: asymptomatic
21	23	M, 62	Alcohol abuse	R: II	A	R: pain	76	R: II	R: asymptomatic
22	24	M, 35	Steroid use	R: II	A	R: pain	65	R: II	R: asymptomatic
23	25	M, 48	Idiopathic	L: II	A	L: discomfort	273	L: II	L: asymptomatic
24	25	M, 48	Idiopathic	R: II	A	R: discomfort	273	R: II	R: asymptomatic
25	26	M, 42	Steroid use	R: II	A	R: pain	103	R: II	R: asymptomatic
26	27	F, 48	Steroid use	R: II	A	R: pain	69	R: II	R: asymptomatic
27	28	F, 71	Steroid use	R: II	A	R: pain	73	R: II	R: asymptomatic
28	29	M, 47	Steroid use	L: II	A	L: pain	64	L: II	L: asymptomatic
29	29	M, 47	Steroid use	R: II	A	R: pain	64	R: II	R: asymptomatic
30	11	F, 65	Idiopathic	L: II	B	L: asymptomatic	68	L: II	L: asymptomatic
31	21	M, 48	Alcohol abuse	L: II	B	L: pain	75	L: II	L: asymptomatic
32	30	M, 43	Steroid use	L: II	B	L: pain	85	L: II	L: asymptomatic

L is short for the left hip, R is short for the right hip.

**TABLE II.** Data on the nine hips who had collapsed at the post-collapse

Hip number	Patient number	Gender, age (years)	Associated condition	ARCO		Symptom at onset	Follow-up time (Months)	ARCO stage at collapse	Comments
				Stage at onset	Type				
33	2	M, 48	Steroid use, alcohol abuse	R: II	B	R: pain	69	R: III	R: discomfort
34	4	M, 53	Steroid use	L: II	B	L: pain	93	L: III	L: asymptomatic
35	4	M, 54	Steroid use	R: II	B	R: asymptomatic	93	R: III	R: asymptomatic
36	10	M, 62	Steroid use	L: II	B	L: pain	88	L: III	L: discomfort
37	11	F, 65	Idiopathic	R: II	B	R: pain	68	R: III	R: asymptomatic
38	12	M, 44	Steroid use	L: II	B	L: asymptomatic	134	L: III	L: asymptomatic
39	24	M, 35	Steroid use	L: II	B	L: pain	65	L: III	L: asymptomatic
40	27	F, 48	Steroid use	L: II	B	L: pain	69	L: III	L: asymptomatic
41	18	F, 55	Steroid use	L: II	A	L: pain	146	L: III	L: asymptomatic

L is short for the left hip, R is short for the right hip, the end point of follow-up is collapse time.

The progression of ONFH is determined by imaging data and physical examination. For the patients who could not come to the hospital for re-examination due to special reasons, we record their hip symptoms by phone and ask them to upload the latest imaging data to us through email.

### Subtype definition of ring-shape sclerotic zone

**Fig. 1.**
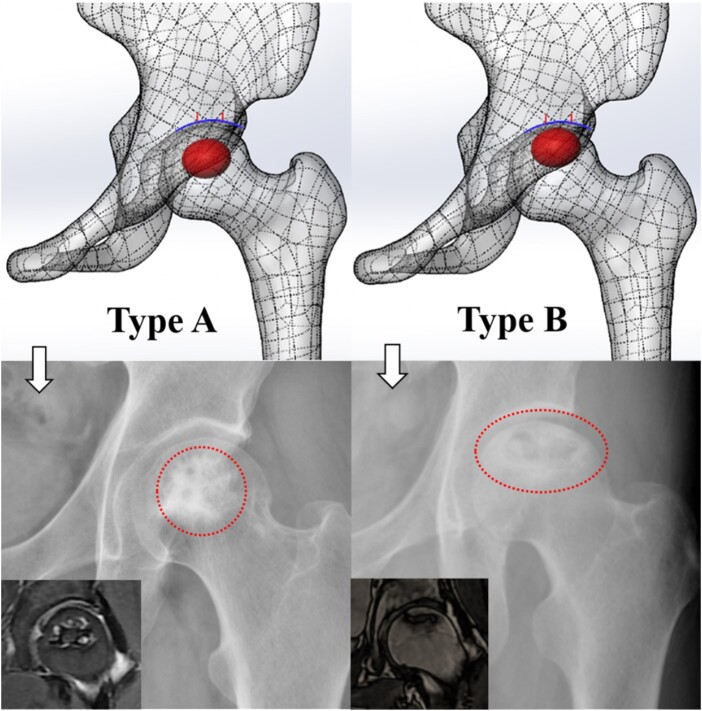
The two subtypes diagram (the hardened zone of type A is at the center of the femoral head; the hardened zone of type B is located in the subchondral bone plate of the femoral head).

In these 30 patients with ring-shaped sclerotic zones, two subtypes were discovered by us from reviewing the patient’s radiographic films. The difference between them is that the sclerotic zone is located at different positions on the femoral head, the sclerotic zone of type A is at the center of the femoral head (being away from the weight-bearing area); the sclerotic zone of type B is located in the subchondral bone plate of the femoral head (within the weight-bearing area) (Fig. 1).

### Data collation

The gender, age, etiology, hip pain or discomfort and follow-up of the ONFH patients are recorded. We list the 32 hips that have not collapsed in Table I, and the nine hips that have collapsed in Table II. (The hips of some of the different cases in Tables I and II may be from different sides of the hips of the same patient.)

### Statistical analysis

Statistical analysis of data is performed with SPSS version 24.0 (IBM Corporation, Armonk, NY, USA). For comparison between two groups, data are analysed by Chi-square test and Kaplan Meier survival curves.

## RESULTS

### General outcomes of the entire population

**Fig. 2.**
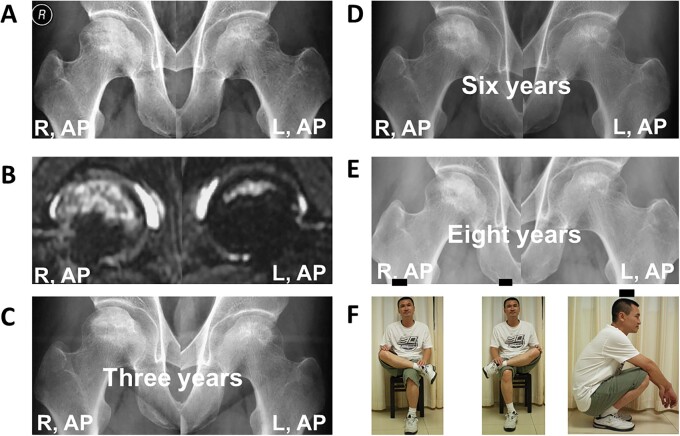
A 50-year-old man (Patient 6) who has idiopathic necrosis of the femoral head. (**A**) Frog-leg lateral radiographs and anteroposterior radiographs show bilateral hips with stage II disease at his initial diagnosis, the right and left are type A. (**B**) MRI image of the suffered hip obtained at initial diagnosis. (**C, D** and **E**) The three years, the sixth year and the eighth year, the patient was free from symptoms and had no radiographic progression of the double femoral head. (F) Until eight years later, the patient was free from symptoms and had good function.

**Fig. 3.**
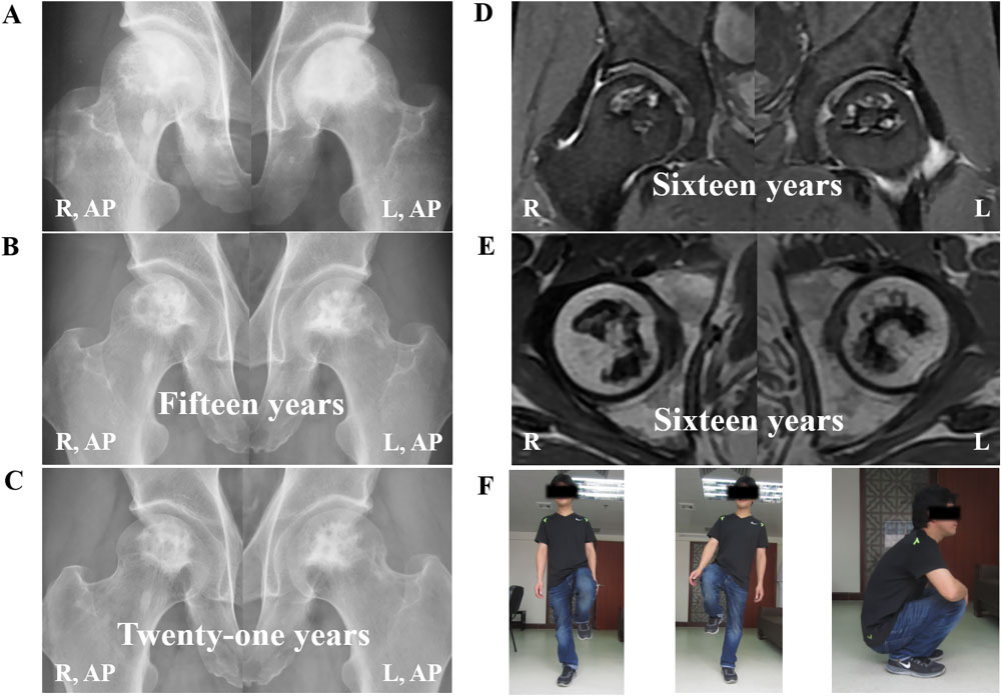
A 48-year-old man (Patient 25) who has idiopathic necrosis of the femoral head. (**A**) Frog-leg lateral radiographs and anteroposterior radiographs show bilateral hips with stage II disease and type A at his initial diagnosis. (**B**) Without special treatment for 15 years, the patient was free from symptoms and had no collapse of the femoral heads bilaterally on frog-leg lateral radiographs and anteroposterior radiographs. (**C**) After 21 years, the patient was free from symptoms and had no radiographic progression of the bilateral femoral head. (**D** and **E**) MRI image of the suffered hip obtained 16 years later. (**F**) At present, the patient was free from symptoms and had good function.

Initially, all the 41 hips have radiographic abnormalities without evidence of collapse (ARCO stage II), 20 (48.8%) of the 41 hips suffered from hip pain, 18 (43.9%) of them are asymptomatic and three (7.3%) of them are discomfort. Without medication or surgery, nine (22.0%) of the 41 hips collapsed, eight of the nine collapsed hips are type B and one is type A. Follow-up to 2019, none of the collapsed hips have apparent collapse progress, and the 30 patients have good daily hip function, 39 (95.1%) of the 41 hips are asymptomatic and only two (4.9%) of them are discomfort (Fig. 2 and 3).

### Different fate of the two subtypes

In the 41 hips, there are 30 hips of type A. Only one of the 30 hips has collapsed, with a collapse rate of 3.3%. Initially, 15 hips are pain or discomfort and 15 hips are asymptomatic. At the latest follow-up, all of them are asymptomatic.

In the 41 hips, there are 11 hips of type B. Eight of the 11 hips have collapsed, with a collapse rate of 72.7%. Initially, eight hips are pain or discomfort and three hips are asymptomatic. At the most recent follow-up, only two are discomfort, the rest hips are asymptomatic.

### Statistical analysis

Chi-square test show that, at first, the occurrence rates of symptomatic hip (pain and discomfort) of type A and type B is no difference (*P* > 0.05). Follow-up to 2019, the occurrence rates of symptomatic hip (pain and discomfort) of type A is significantly lower than that of type B and differences between these two groups are significant (*P* < 0.05).

**Fig. 4.**
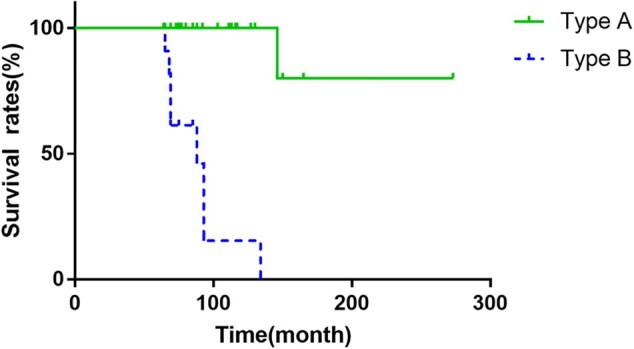
The time of first occurrence of collapse was used as the survival curve of the end point.

Kaplan Meier survival curve analysis show that the mean survival time of the two subtypes is 192.770 M (95% CI: 147.819 ∼ 237.721 M). The mean survival time of type A is 247.600 M (95% CI: 203.072 ∼ 292.128 M) and type B is 88.795 M (95% CI: 72.607 ∼ 104.984 M). The survival rate of femoral head of type A is significantly higher than that of type B (*P* < 0.005, Fig. 4).

## DISCUSSION

The most striking finding of this study is that the ARCO stage-II osteonecrosis of femoral head (ONFH) which the sclerotic zone is ring-shaped usually has an ideal prognosis. According to the anteroposterior view and frog-leg lateral view radiographs, surprisingly, we find two subtypes, one is type A in which the ring-shaped sclerotic zone located in the middle of the femoral head (being away from the weight-bearing area), another is type B in which the ring-shaped sclerotic zone located in the subchondral bone plate of the femoral head (within the weight-bearing area). Type A has an extremely low rate of collapse (3.3%), and the symptoms usually resolve themselves within a certain period of time. Type B has a high rate of collapse (72.7%). Fortunately, the progress of collapse is slow, and the symptoms of type B hip are not obvious, which has no significant impact on the daily life of patients.

There are 23 hips (56.1%) of the 41 hips with pain or discomfort in the hip initially; however, in the last follow-up, only two hips (4.9%) of the 41 hips were uncomfortable. Follow-up to 2019, the occurrence rates of symptomatic hip (pain and discomfort) of type A is significantly lower than that of type B (*P* < 0 .05). Because such patients with ONFH are generally asymptomatic and have good joint function, no matter whether the collapse occurs or not, it does not affect the daily life of the patient. Therefore, many patients are reluctant to return to the hospital for follow-up, and we can only conduct telephone follow-up of them, which is also a shortcoming of this study.

In our series (average follow-up 104 months, at least 64 months in each case), 32 hips (78.0%) of the 41 hips had not collapsed so far. Although there is no consensus among international experts, JIC is generally regarded as the most reliable classification and prediction method. A previous study showed that the 10-year collapse rates of different Japanese Investigation Committee (JIC) classification were significant differences. Type A was 0%, type B was 6%, type C1 was 68% and type C2 was 82% [43]. Surprisingly, one of the ring-shaped sclerotic zone subtypes, type A described in this paper has the same low risk of collapse as type A ONFH in JIC classification. We suspect that the reason is the ring-shaped sclerotic zone of type A being away from the load area, moreover, the condensed trabecular bone in the ring-shaped sclerotic zone protect necrotic tissue which maintain the mechanical strength during the bone repair process [29–31].

When ONFH occurs, the weight-bearing capacity of the femoral head is weakened, and the greater the mechanical force acting directly on the necrotic bone mass, the more likely it is to collapse. We suspect that the lower risk of collapse in such cases is most of the mechanical stress is assumed to be exerted on the surviving bone tissue while the patient was standing or walking. In addition, of the 41 cases, nine hips (22.0%) have collapsed, eight of them have a ring sclerotic zone located in the weight-bearing area of the femoral head and one case located in the middle of femoral head. We speculate that the cause of the collapse is because the necrotic area is located on the weight-bearing surface of the femoral head. When the patient stands and walks, the necrotic bone is subjected to a large pressure, which causes collapse. Therefore, under the same load, the survival rate of femoral head of type A is significantly higher than that of type B (*P* < 0.005).

## CONCLUSIONS

This study demonstrates that the ARCO stage II ONFH with ring-shaped sclerotic zone at the center of the femoral head (being away from the weight-bearing area), which is defined as type A by our team, has lower collapse risk than in the subchondral bone plate of the femoral head (within the weight-bearing area), which is defined as type B by our team. Generally, type A shows a more satisfactory clinical outcomes in a mid-term follow-up.

## FUNDING

The National Natural Science Foundation of China (GenERαl Projects), No. 81473697, 81873327 (to HW); Guangdong Provincial Science and Technology Department-Guangdong Academy of Chinese Medicine Joint Research Special Project, No. 2016A020226028 (to WQS); Guangdong Natural Science Foundation, No. 2017A030313698 (to WQS).

## CONFLICT OF INTEREST STATEMENT

None declared.
